# Supplementary material for: Predicting visual function by interpreting a neuronal wiring diagram
Source: Nature. 2024 Oct 2;634(8032):113–23. doi: 10.1038/s41586-024-07953-5 (PMC11446822; doi:10.1038/s41586-024-07953-5)
Supplement: Supplementary file 6 — CRF and ERF predictions for individual TmY4 and TmY9 cells. Analogous to Supplementary Data 3, but for TmY target types. Shown are the top four monosynaptic pathways, the strongest pathway passing through each of the top ten intermediary types (ranking from Extended Data Fig. 7), and the trisynaptic pathway Tm1–TmY–Dm3–TmY (see the section entitled Prediction of spatial normalization). [file 41586_2024_7953_MOESM6_ESM.zip › DataS4/TmY9q/720575940622722544.pdf]

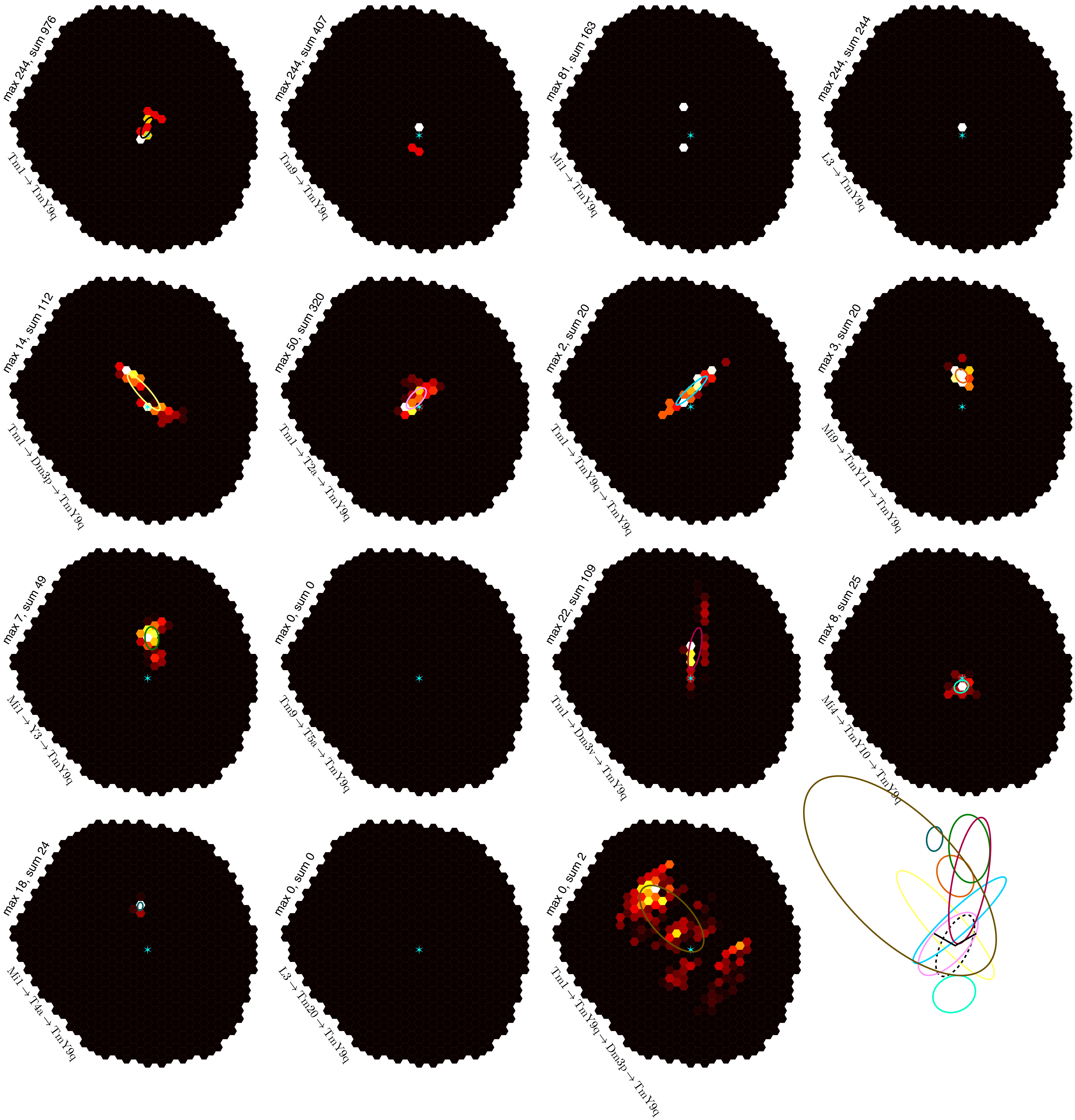
max 244, sum 976
Tm1 → TmY9q
max 244, sum 407
Tm9 → TmY9q
max 81, sum 163
Mi1 → TmY9q
max 244, sum 244
L3 → TmY9q
max 14, sum 112
Tm1 → Dm3p → TmY9q
max 50, sum 320
Tm1 → T2a → TmY9q
max 2, sum 20
Tm1 → TmY9q → TmY9q
max 3, sum 20
Mi9 → TmY11 → TmY9q
max 7, sum 49
Mi1 → Y3 → TmY9q
max 0, sum 0
Tm9 → T5a → TmY9q
max 22, sum 109
Tm1 → Dm3v → TmY9q
max 8, sum 25
Mi4 → TmY10 → TmY9q
max 18, sum 24
Mi1 → T4a → TmY9q
max 0, sum 0
L3 → Tm20 → TmY9q
max 0, sum 2
Tm1 → TmY9q → Dm3p → TmY9q
